# Interactions among genes involved in reverse cholesterol transport and in the response to environmental factors in dyslipidemia in subjects from the Xinjiang rural area

**DOI:** 10.1371/journal.pone.0196042

**Published:** 2018-05-14

**Authors:** Xinping Wang, Heng Guo, Yu Li, Haixia Wang, Jia He, Lati Mu, Yunhua Hu, Jiaolong Ma, Yizhong Yan, Shugang Li, Yusong Ding, Mei Zhang, Qiang Niu, Jiaming Liu, Jingyu Zhang, Rulin Ma, Shuxia Guo

**Affiliations:** 1 Department of Public Health, Shihezi University School of Medicine, Shihezi, China; 2 Key Laboratory of Xinjiang Endemic and Ethnic Diseases of the Ministry of Education, Shihezi University School of Medicine, Shihezi, China; South Australian Health and Medical Research Institute, AUSTRALIA

## Abstract

Gene-gene and gene-environment interactions may be partially responsible for dyslipidemia, but studies investigating interactions in the reverse cholesterol transport system (RCT) are limited. We explored these interactions in a Xinjiang rural population by genotyping five SNPs using SNPShot technique in APOA1, ABCA1, and LCAT, which are involved in the RCT (690 patients, 743 controls). We conducted unconditional logistical regression analysis to evaluate associations and generalized multifactor dimensionality reduction to evaluate interactions. Results revealed significant differences in rs670 and rs2292318 allele frequencies between cases and controls (*P*<0.025). rs670 G allele carriers were more likely to develop dyslipidemia than A allele carriers (OR = 1.315, OR 95% CI: 1.067–2.620; *P* = 0.010). rs2292318 T allele carriers were more likely to develop dyslipidemia than A allele carriers (OR = 1.264, OR 95% CI: 1.037–1.541; *P* = 0.020). Gene-gene interaction model APOA1rs670-ABCA1rs1800976-ABCA1rs4149313-LCATrs1109166 (*P* = 0.0107) and gene-environment interaction model ABCA1rs1800976-ABCA1rs4149313-LCATrs1109166-obesity-smoking were optimal dyslipidemia predictors (*P* = 0.0107) and can interact (4). Differences in A-C-A-C-A and G-G-G-T-G haplotype frequencies were observed (*P*<0.05). Serum lipid profiles could be partly attributed to RCT gene polymorphisms. Thus, dyslipidemia is influenced by APOA1, ABCA1, LCAT, environmental factors, and their interactions.

## Introduction

Coronary heart disease and cerebrovascular disease are common public health problems in both high- and low-income countries [1], Dyslipidemia is an important risk factor for these diseases [2], and is characterized by elevated levels of triglycerides (TG), total cholesterol (TC), and low-density lipoprotein cholesterol (LDL-C) and reduced levels of high-density lipoprotein cholesterol (HDL-C). Dyslipidemic individuals have also been reported to have higher risk of coronary artery disease [3–6].

Dyslipidemia is a complex human disease that is influenced by both genetic and environmental factors [7]. Understanding the risk of developing complex diseases in a population involves studying a variety of factors. In particular, genetic and environmental factors interact to influence disease risk. Gene-gene interactions or gene-environment interactions are believed to produce combined effects in one or more genes or with one or more environmental factors that cannot be fully explained by their separate marginal effects [8]. Therefore, identification of gene-gene and gene-environment interactions will improve understanding of missing heritability [9, 10].

Reverse cholesterol transport (RCT) is the process by which cholesterol is effluxed from peripheral tissues through acceptor particles, primarily HDL, to the plasma for subsequent uptake by the liver. RCT involves numerous lipid transfer proteins, enzymes, apolipoproteins, and membrane-bound receptors [11, 12]. ATP-binding cassette A1 (ABCA1), apolipoprotein A-1 (APOA-1), and cholesterol acyltransferase (LCAT) are known to play crucial roles in the RCT system.

APOA1, ABCA1 and LCAT that encode these proteins, as well as genes responsible for regulating their transcription, are candidates for influencing variation in plasma lipid levels. Mutations in the APOA1, ABCA1, and LCAT can influence transcription and translation, leading to abnormal lipid metabolism [13–15]. Recent studies have suggested the relationship between lipid levels and APOA1, ABCA1, and LCAT polymorphisms; however, results remain inconsistent across different races [16–22]. One major reason for the observed inconsistency is that different studies focused on the association of single gene polymorphisms of APOA1, ABCA1, or LCAT and risk factors with lipid levels [13–15, 20, 21, 23–26], However, abnormal blood lipid profiles are caused by multiple genes and environmental factors, each of which can contribute a minor marginal effect. Based on previous observations, we hypothesized that dyslipidemia is jointly caused by the APOA1, ABCA1, and LCAT genes and their interactions with environmental factors.

Therefore, to explore gene-gene and gene-environment interactions underlying dyslipidemia, we investigated five (rs670, rs1800976, rs4149313, rs2292318, rs1109166) single nucleotide polymorphisms (SNPs) located in APOA1, ABCA1, and LCAT based on a model RCT pathway. The study population consisted of 1,433 individuals comprising 690 dyslipidemic subjects and 743 normal controls. In addition, we investigated the relationship between APOA1, ABCA1, and LCAT gene variants and serum lipid levels.

## Materials and methods

### Study population

The present study was conducted between March 2009 and February 2010. A total of 3,049 subjects aged 18 years and above who were residents of Jiashi County and 5,692 subjects aged 18 years and above who were residents of Yili region Xinyuan County of Xinjiang Kashgar Prefecture were selected using the stratifying cluster sampling method. Xinyuan County and Jiashi County are at a distance of ~3692 km (2294 miles) and ~4329 km (2690 miles) from Beijing, respectively. A total of 743 dyslipidemic patients were assigned to the case group, and 690 subjects with normal blood lipid levels were assigned to the control group. The majority of participants in the control group were age- and sex-matched to those in the case group at an approximate ratio of 1:1. Subjects with hypertension, diabetes, liver diseases, renal diseases, or malignant tumors were excluded from the study.

The protocol was approved by the Institutional Ethics Review Board (IERB) of the First Affiliated Hospital of Shihezi University School of Medicine (IERB No. SHZ2010LL01). Written informed consent was obtained from each participant.

### Epidemiological survey and biochemical index determination

All study subjects completed a demographic information survey questionnaire, which included details on past and/or current disease(s), family history, smoking and drinking habits, diet, and physical exercise status during face-to-face interview. Blood pressure, height, weight, waist circumference and hip circumference were measured according to standardized methods [27].

All blood samples were tested for various biochemical parameters according to the 2007 China Adult Dyslipidemia Prevention Guide [28] of dyslipidemia. Laboratory analyses of blood samples included tests for fasting TG, LDL-C, TC, HDL-C, and fasting plasma glucose (FPG) levels, all of which were analyzed using an automatic biochemical analyzer (AU400, Olympus: Tokyo, Japan).

### Diagnostic criteria

According to the China Adult Dyslipidemia Prevention Guide (2007) [28], dyslipidemia is defined as the presentation of any one or more of the following four items: (1) TG≥2.26 mmol/L (200 mg/dL); (2) HDL-C<1.04 mmol/L (40 mg/dL); (3) LDL-C≥4.14 mmol/L (160 mg/dL); and (4) TC≥ 6.22 mmol/L (240 mg/dL). The drinking rate was calculated by dividing the number of drinkers by the total number of participants in each group.

The Working Group on Obesity in China defines obesity as having BMI ≥28 kg/m2[29, 30].

### DNA extraction and genotyping analysis

Fasting venous blood (200 μL) was taken from each study subject, and a blood genomic DNA isolation kit (Non-centrifugal columnar, Tiangen, Beijing, China) was used to isolate the genomic DNA. The extracted DNA was verified by gel electrophoresis (0.7% agarose). A NanoDrop spectrophotometer (Thermo Scientific, Waltham, MA, USA) was used for quantitative determination of DNA concentration and purity: concentration ≥30 ng/μL and purity levels (optical density [OD]: OD260/OD280) of 1.7–2.0 were considered acceptable. Samples that met these criteria were diluted to 10–30 ng/μL using double-distilled water and then stored at -80 °C. The process of polymerase chain reaction (PCR) amplification, purification, and single-base extension were depicted in details in our published paper [31]and the DOI link dx.doi.org/10.17504/protocols.io.mmtc46n. All representative SNP genotyping experiments were performed using TaqMan technology on an ABI3730xl system (Applied Biosystems, Foster City, CA, USA). ABI GeneMapper was used to complete the classification and present the results.

For the five SNPs, the success rates were all 100%, and no evidence of departure from Hardy-Weinberg equilibrium was observed in control and case subjects using chi-squared-test analysis.

### Statistical analysis

Epidata 3.02 software (EpiData Association, Odense, Denmark) was used to establish a database, and the double entry method was used for data input and logic error detection.

Descriptive statistics, t-test or Mann Whitney test, and ANOVA or Kruskal Wallis tests were performed as appropriate after checking for normality by Kolmogorov-Smirnov test, the chi-square test was used to evaluate differences between groups for the categorical variables. The gene counting method was used to calculate genotype and allele frequencies. The chi-square test was used to test for Hardy-Weinberg equilibrium. Unconditional logistical regression analysis was conducted to calculate the odds ratio (OR) and 95% confidence interval (95% CI) with adjustments for age and sex. Gene-gene and gene-environment interactions were identified using GMDR0.9 [32] which is based on the score of a generalized linear model and allows the adjustment of discrete and quantitative covariates, as well as applicable to both dichotomous and continuous data; in this study, ten-fold cross validation was performed. The confounding factors, namely, age, sex, height, weight, and drinking, were included as covariates for interaction analysis. SHEsis software was used to analyze haplotypes [33]. Statistical significance was considered at *P*<0.05. The significance threshold was adjusted for multiple comparison tests according to Bonferroni correction, and set at *P*<0.025 (0.05/2 = 0.025) when apply multiple comparison.

## Results

### General comparison of study subjects

The characteristics of the study individuals are provided in Table 1. There were significant differences in characteristics, such as height, weight, BMI, waist circumference, hip circumference and waist-to-hip ratio, as well as lipid profiles. Moreover, the case group had higher mean blood pressure levels compared to the control group (*P*<0.05), and the rates of obesity and smoking were lower in the control group than in the case group (*P*<0.001). No differences in drinking rates were detected between the two groups (*P*>0.05).

**Table 1:** Comparison of general characteristics and serum lipids levels between the control and case groups.

Characteristic	Control(n = 743)	Case(n = 690)	*P*
**male/female**	366/377	342/348	0.908
**Age(year)**	43.49±15.35	45.10±15.09	0.061
**Height(cm)**	161.09±8.78	162.09±9.04	0.031
**Weight(kg)**	58.73±10.95	66.89±13.85	<0.001
**Body Mass Indes, kg/m2**	22.56±3.47	25.35±4.21	<0.001
**Waist Circumference, cm**	81.21±9.89	89.94±11.90	<0.001
**Hip Circumference**	93.86±7.61	98.56±8.50	<0.001
**Waist-to-Hip Ratio**	0.86±0.07	0.91±0.08	<0.001
**TG (mmol/l)**	0.79±0.28	2.17±1.69	<0.001
**TC (mmol/l)**	3.99±0.71	4.90±1.58	<0.001
**LDL-C (mmol/l)**	2.02±0.53	2.73±1.07	<0.001
**HDL-C (mmol/l)**	1.45±0.29	1.08±0.41	<0.001
**Systolic blood pressure,mmHg**	128.39±22.94	133.51±23.56	<0.001
**Distolic blood pressure,mmHg**	80.72±13.74	84.07±14.51	<0.001
**Obesity, n(%)**	60(8.1)	166(24.1)	<0.001
**smoking, n(%)**	176(23.7)	175(25.4)	0.045
**drinking, n(%)**	48(6.5)	41/(5.9)	0.954

TG, triglyceride; TC, total cholesterol; LDL-C, low-density lipoprotein-cholesterol; HDL-C, high-density lipoprotein cholesterol.

Values are presented either mean±SD or n (%). *t*-test or Wilcoxon rank sum test was used to obtained the P value for continuous variables, and a chi-square test was used to obtain significance for categorical variables, *P*<0.05 significant.

### Genotype and allele frequencies

A comparison of the genotype and allele frequencies of the five SNPs between the control and case groups are presented in Table 2. No significant differences in the genotype and allele frequencies of ABCA1rs1800976, ABCA1rs4149313 and LCATrs1109166 were observed (*P*>0.025). Compared with carriers of the APOA1rs670 A alleles, G allele carriers were significantly more likely to develop dyslipidemia (OR = 1.315, OR 95% CI: 1.067–2.620; *P* = 0.010). Carriers of the LCATrs2292318 T allele were more likely to develop dyslipidemia than A allele carriers (OR = 1.264, 95% CI: 1.037–1.541; *P* = 0.020).

**Table 2:** Genotypic and allelic frequencies between the control and case groups.

Gene	SNPs	Genotype/allele	control	case	*P*	OR(95%CI)
**APOA1**	**rs670**	**AA**	12(1.62)	18(2.60)	0.038	
		**AG**	166(22.34)	187(27.10)	0.14	1.747(0.833–3.664)
		**GG**	565(76.04)	485(70.30)	0.028	1.312(1.031–1.671)
		**G**	1296(87.21)	1157(83.85)	0.010*	1.315(1.067–2.620)
		**HWE-*P***	0.996	0.961		
**ABCA1**	**rs1800976**	**CC**	150(20.19)	147(21.30)	0.855	
		**CG**	359(48.32)	332(48.12)	0.579	1.087(0.910–1.458)
		**GG**	234(31.49)	211(30.58)	0.835	1.026(0.808–1.302)
		**G**	827(55.65)	754(54.64)	0.585	1.041(0.899–1.207)
		**HWE-*P***	0.441	0.565		
	**rs4149313**	**AA**	193(25.98)	197(28.55)	0.459	
		**AG**	372(50.07)	342(49.57)	0.217	1.203(0.897–1.614)
		**GG**	178(23.96)	151(21.88)	0.547	1.084(0.834–1.408)
		**G**	728(48.99)	644(46.67)	0.213	1.098(0.948–1.271)
		**HWE-*P***	0.911	0.962		
**LCAT**	**rs2292318**	**CC**	492(66.22)	497(72.03)	0.057	
		**CT**	231(31.09)	179(25.94)	0.3	1.443(0.721–2.889)
		**TT**	20(2.69)	14(2.03)	0.779	1.107(0.544–2.252)
		**T**	271(18.24)	207(15.00)	0.020*	1.264(1.037–1.541)
		**HWE-*P***	0.649	0.246		
	**rs1109166**	**AA**	454(61.10)	461(66.80)	0.08	
		**AG**	262(35.26)	207(30.00)	0.455	1.246(0.699–2.221)
		**GG**	27(3.63)	22(3.20)	0.919	0.970(0.537–1.752)
		**G**	316(21.26)	251(18.20)	0.039	1.215(1.009–1.461)
		**HWE-*P***	0.833	0.148		

HWE-*P*: Hardy-Weinberg equilibrium *P* value. rs670-AA, rs1800976-CC and rs4149313-AA genotypes used as a reference genotype for obtaining the Odds Ratio calculations separated for each single nucleotide polymorphism.

Binary logistical regression analysis was used to obtained *P* value, the odds ratio (OR) and 95% confidence interval (95% CI) to obtained the SNPs difference between case and control group with adjustments for age and sex.

*P<0.025 significant.

### The genotypes of five SNPs and serum lipid profiles in normal and dyslipidemic individuals

Table 3 shows genotypes of five SNPs and serum lipid profiles which presented with median and interquartile range (25th, 75th percentile) and adjusted for sex and age in normal and dyslipidemic individuals. In the control group, carriers of the rs670 AA genotype showed higher TG levels than those of AG and GG genotype carriers (*P* = 0.014). Individuals with the rs2292318 TT genotype showed higher TG levels than those with the CT and CC genotypes in normal individuals (*P* = 0.003). For the remaining loci, control and case groups showed no significant differences in the genotype distributions and TG, TC, LDL-C, and HDL-C levels.

**Table 3:** The genotypes of five SNPs and serum lipid profiles (mmol/L) in normal and dyslipidemic individuals.

SNPs	Group	Genotype	TG	TC	LDL-C	HDL-C
**rs670**	**control**	**AA**	0.95(0.86–1.09) *	4.33(4.05–4.74)	2.33(1.99–2.50)	1.41(1.21–1.50)
		**AG**	0.79(0.61–0.98) *	4.11(3.49–4.58)	1.96(1.60–2.51)	1.41(1.25–1.60)
		**GG**	0.77(0.59–0.95) *	4.02(3.62–4.43)	1.94(1.65–2.37)	1.40(1.24–1.62)
	**case**	**AA**	2.02(1.57–2.89)	4.68(3.54–5.86)	2.42(1.68–3.58)	0.92(0.88–0.98)
		**AG**	2.18(1.13–2.80)	4.96(3.75–6.24)	2.66(1.87–3.36)	0.95(0.82–1.17)
		**GG**	1.93(1.18–2.68)	4.64(3.82–6.13)	2.63(1.97–3.40)	0.97(0.85–1.21)
**rs1800976**	**control**	**CC**	0.77(0.62–0.98)	4.04(3.47–4.45)	1.91(1.55–2.32)	1.39(1.25–1.60)
		**CG**	0.78(0.61–0.97)	4.11(3.65–4.54)	2.00(1.70–2.46)	1.42(1.25–1.64)
		**GG**	0.76(0.58–0.91)	4.00(3.55–4.38)	1.94(1.67–2.33)	1.38(1.23–1.57)
	**case**	**CC**	1.76(1.09–2.50)	4.91(3.70–6.26)	2.67(1.93–3.42)	0.9(0.86–1.20)
		**CG**	2.17(1.25–2.78)	4.73(3.85–6.13)	2.63(2.00–3.48)	0.96(0.83–1.22)
		**GG**	1.98(1.19–2.68)	4.63(3.76–5.85)	2.63(1.91–3.29)	0.96(0.84–1.18)
**rs4149313**	**control**	**AA**	0.77(0.61–0.96)	4.07(3.56–4.52)	1.97(1.64–2.47)	1.41(1.23–1.64)
		**AG**	0.78(0.59–0.96)	4.08(3.62–4.48)	1.95(1.68–2.35)	1.39(1.25–1.58)
		**GG**	0.78(0.63–0.94)	4.01(3.53–4.45)	1.94(1.63–2.39)	1.43(1.24–1.63)
	**case**	**AA**	2.10(1.34–2.77)	4.64(3.78–5.93)	2.54(2.01–3.38)	0.96(0.84–1.19)
		**AG**	1.91(1.13–2.69)	4.70(3.84–6.22)	2.70(1.94–3.36)	0.97(0.83–1.21)
		**GG**	2.02(1.14–2.68)	4.79(3.70–6.21)	2.56(1.89–3.49)	0.96(0.87–1.16)
**rs2292318**	**control**	**CC**	0.75(0.59–0.93) *	4.03(3.58–4.47)	1.93(1.64–2.37)	1.43(1.25–1.63)
		**CT**	0.80(0.61–1.03) *	4.14(3.60–4.51)	2.02(1.67–2.46)	1.36(1.23–1.56)
		**TT**	0.87(0.76–1.11) *	4.08(3.77–4.60)	2.01(1.81–2.18)	1.34(1.21–1.55)
	**case**	**CC**	2.01(1.17–2.70)	4.90(3.80–6.29)	2.69(1.97–3.54)	0.97(0.84–1.23)
		**CT**	1.89(1.14–2.69)	4.47(3.74–5.64)	2.39(1.88–3.23)	0.95(0.83–1.11)
		**TT**	2.45(1.18–3.04)	4.37(4.10–5.46)	2.53(2.00–3.34)	1.01(0.89–1.14)
**rs1109166**	**control**	**AA**	0.76(0.60–0.94)	4.03(3.58–4.48)	1.92(1.65–2.36)	1.43(1.24–1.63)
		**AG**	0.80(0.59–0.98)	4.08(3.60–4.48)	2.03(1.64–2.47)	1.38(1.23–1.58)
		**GG**	0.84(0.62–1.12)	4.15(3.79–4.61)	1.89(1.67–2.17)	1.32(1.24–1.55)
	**case**	**AA**	2.02(1.17–2.72)	4.92(3.78–6.29)	2.69(1.98–3.48)	0.97(0.84–1.23)
		**AG**	1.89(1.17–2.68)	4.55(3.76–5.75)	2.43(1.89–3.31)	0.96(0.83–1.18)
		**GG**	2.02(1.18–3.04)	4.32(4.02–5.21)	2.35(2.00–3.07)	0.95(0.77–1.03)

TC: Total Cholesterol; TG: triglyceride; HDL-C: high-density lipoprotein-cholesterol; LDL-C: low-density lipoprotein-cholesterol;

*P<0.025.

Lipid levels were presented with median and interquartile range (25th, 75th percentile). Kruskal Wallis test was used to obtained *P* value.

### Association of gene-gene and gene-environment interactions with dyslipidemia

Table 4 summarizes the results from the GMDR (Generalized multifactor dimensionality reduction) analysis of the association of gene-gene and gene-environment interactions with dyslipidemia. GMDR was used to assess gene-gene interactions of five SNPs located in the APOA1, ABCA1, and LCAT genes with adjustments for age, sex, smoking and drinking status, as well as blood pressure. After adjusting for co-variables, the best combination was determined to be APOA1rs670-ABCA1rs1800976-ABCA1rs4149313-LCATrs1109166, suggesting that these four variants jointly contributed to the etiology of dyslipidemia. For the gene-environment analysis, which included age, sex, and blood pressure as the covariates, GMDR identified the five-factor interaction model of ABCA1rs1800976-ABCA1 rs4149313-LCATrs1109166-obesity-smoking as the best model for dyslipidemia, with a testing balance accuracy of 0.5313 and a maximum cross-validation consistency of 10/10 (*P* = 0.0107).

**Table 4:** Association of gene-gene and gene-environment interactions with dyslipidemia.

Model	training balance accurary	testing balance accurary	CV consistency	*P*
**Gene-Gene**				
**rs670**	0.5316	0.5111	5/10	0.1719
**rs670-rs1109166**	0.5436	0.5036	5/10	0.0547
**rs670-rs1800976-rs4149313**	0.5578	0.4900	3/10	0.8281
**rs670-rs1800976-rs4149313-rs1109166**	0.5827	0.5359	10/10	0.0107*
**rs670-rs1800976-rs4149313-rs2292318-rs1109166**	0.5932	0.5263	10/10	0.1719
**Gene-Environment**				
**rs1800976**	0.5321	0.5032	6/10	0.8281
**rs1800976-rs1109166**	0.5492	0.4979	4/10	0.6230
**rs4149313-rs1109166-obesity**	0.5708	0.4992	5/10	0.1719
**rs1800976-rs4149313-rs1109166-obesity**	0.6015	0.5053	9/10	0.6230
**rs1800976-rs4149313-rs1109166-obesity-smoking**	0.6459	0.5313	10/10	0.0107*
**rs670-rs1800976-rs4149313-rs1109166-obesity-smoking**	0.6787	0.4986	8/10	0.6230

*P<0.05, Generalized multifactor dimensionality reduction analysis was used to obtained *P* value

### Haplotype analysis

Haplotype analysis results for the five SNPs are shown in Table 5. Global haplotype frequencies were significantly different between the control and case groups (P<0.001). The case group had significantly higher frequency of the A-C-A-C-A haplotype than the control group, whereas the G-G-G-T-G haplotype was less frequent in the case group than in the control group (*P*<0.05).

**Table 5:** Comparison of haplotype frequencies of five SNPs located in the ABCA1, APOA1, and LCAT genes between the case and control groups.

Haplotype	Case, n (%)	Control, n (%)	*χ*^*2*^	Fisher’s *P*	Pearson’s *P*	OR(95CI)
**A-C-A-C-A**	48.03(0.035)	25.71(0.017)	8.510	0.004	0.004*	2.030(1.250–3.297)
**A-C-G-C-A**	47.45(0.034)	36.25(0.024)	2.384	0.123	0.123	1.411(0.910–2.189)
**A-G-A-C-A**	30.18(0.022)	48.56(0.033)	3.286	0.070	0.070	0.655(0.413–1.038)
**A-G-G-C-A**	54.84(0.040)	39.15(0.026)	3.866	0.049	0.049	1.516(0.999–2.300)
**G-C-A-C-A**	226.84(0.164)	242.78(0.163)	0.002	0.967	0.967	0.996(0.816–1.215)
**G-C-A-T-G**	38.01(0.028)	53.21(0.036)	1.708	0.191	0.191	0.755(0.494–1.152)
**G-C-G-C-A**	192.01(0.139)	217.34(0.146)	0.424	0.515	0.515	0.932(0.755–1.151)
**G-C-G-T-G**	39.10(0.028)	37.81(0.025)	0.191	0.663	0.663	1.106(0.703–1.741)
**G-G-A-C-A**	292.40(0.212)	275.89(0.186)	2.699	0.100	0.100	1.168(0.970–1.405)
**G-G-A-C-G**	23.98(0.017)	13.45(0.009)	3.736	0.053	0.053	1.919(0.980–3.757)
**G-G-A-T-G**	47.35(0.034)	65.73(0.044)	2.007	0.157	0.157	0.760(0.519–1.112)
**G-G-G-C-A**	233.12(0.169)	276.01(0.186)	1.698	0.193	0.192	0.879(0.725–1.067)
**G-G-G-T-G**	43.60(0.032)	72.68(0.049)	5.774	0.016	0.016*	0.627(0.428–0.920)

Haplotypes of five SNPs in the following order (from left to right): rs670 (A>G), rs1800976 (C>G), rs4149313

(A>G), rs12292318 (C> T), rs1109166 (A > G).

* *P*<0.05.

The haplotype frequencies below 0.01 are not included in the table, global *P* < 0.001; A powerful software platform SHEsis was used to obtained *P* value.

## Discussion

Dyslipidemia is a complex human disease that is influenced by both genetic and environmental factors [7], Interactions among multiple genetic and environmental factors are known to exert joint effects, which serve as an important biological basis for understanding complex diseases and phenotype variation [34–37]. Recent studies have now focused on detecting factors that, owing to their interactions with other genetic (or environmental) factors, cannot be identified using standard single-locus tests. Thus, unraveling the so-called “missing heritability” [38], that is not limited to single-marker analysis will facilitate understanding of the mechanisms by which genetic and environmental factors contribute to the formation of complex traits. In the current study, we explored associations of APOA1, ABCA1, and LCAT genes with dyslipidemia and focused on gene polymorphisms involved in the RCT system. A total of 690 patients and 743 control subjects were analyzed to evaluate gene-gene and gene-environment interactions between dyslipidemia and single-nucleotide polymorphisms (SNPs) that influence lipid metabolism and to elucidate the mechanisms underlying missing heritability. In this study, we investigated the contribution of SNPs from RCT-related genes to susceptibility. APOA1rs670 and LCATrs2292318 polymorphisms are associated with dyslipidemia risk. Gene-gene interaction of APOA1rs670-ABCA1rs1800976-ABCA1rs4149313-LCATrs1109166 and the gene-environment interaction of ABCA1rs1800976-ABCA1rs4149313-LCATrs1109166-obesity-smoking were identified based on GMDR analysis. Our findings suggested that the interaction among APOA1, ABCA1, LCAT conferred the genetic susceptibility to dyslipidemia in Xinjiang Rural Area. We consider the present study to be explorative and hypothesis-generating and thus find the results interesting. However, a more convincing conclusion can only be reached by further independent replication studies.

APOA-1, ABCA1, and LCAT play important roles in the RCT system, as well as in lipid metabolism. APOA-I functions as a cholesterol acceptor, and APOA1 variants are known to be associate with lipid levels. ABCA1 transports cellular cholesterol across the cell membrane to plasma acceptor particles, such as APOA-I [39, 40], LCAT converts unesterified cholesterol to cholesterol esters during the process of HDL maturation [14]. Mutations in APOA-1, ABCA1, and LCAT can cause alterations and protein transcription and translation, eventually leading to abnormal lipid metabolism [13–15].

Dyslipidemia is determined by a plurality of factors. In the present study, we found that anthropometric characteristics, such as height, weight, BMI, waist circumference, hip circumference and waist-to-hip ratio were significantly higher in the dyslipidemia case group than in the control group. Furthermore, the two groups showed significant differences in blood pressure measurements, consistent with the results of previous study in a different population, in which an association between blood pressure and serum lipid levels was stably observed [41]. Furthermore, individuals in the case group had higher incidence of obesity than those in the control group. On the other hand, our findings were consistent with other reports, in which obesity based on waist circumference and weight was significantly higher in cases than controls [23–25]. In addition, the smoking rate was lower in the control group than the case group, which indicated that lifestyle factors, such as smoking, were associated with dyslipidemia. The above findings are consistent with the results of a previous study [42], that detected no differences in drinking habits between the two groups. This implies that drinking status is not the primary driver of abnormal blood lipids levels, which is inconsistent with the results reported by Rao et al. [43].

Our study showed that the genotype frequencies were in Hardy-Weinberg equilibrium and can represent the target population. Allelic frequencies of the five target SNPs also varied among different races in the NCBI database (URL: https://www.ncbi.nlm.nih.gov/snp/), indicating significant racial/ethnic variation in allelic frequencies in the APOA1, and LCAT genes. Results also showed significant differences in the distributions of rs670 and rs2292318 between the control and case groups. For rs670, G allele carriers were significantly more likely to develop dyslipidemia. Carriers of the rs2292318 T allele were more likely to develop dyslipidemia than A allele carriers. These results suggest an association between APOA1 and LCAT polymorphisms and dyslipidemia.

Previous studies have demonstrated the association of polymorphisms in APOA1, ABCA1, and LCAT with serum lipid levels in humans. However, the obtained data remain inconsistent. In previous reports, the APOA1 rs670 polymorphism was found to be correlated with dyslipidemia [22] and HDL-C levels [20, 21]. The ABCA1 rs1800976 polymorphism was associated with HDL-C levels in the Suita population, but a different study found no association between ABCA1 rs1800976 genotype and lipid levels [18, 19]. The ABCA1 rs4149313 polymorphism showed favorable effects on HDL-C levels [44, 45]; Significant associations were observed between LCAT rs2292318 polymorphisms and diabetic dyslipidemia [17], whereas the association between LCAT rs2292318 and HDL cholesterol was not statistically significant in a sample population of French Canadian ancestry [16]. In the present study, we demonstrated an association between the APOA1, ABCA1, and LCAT polymorphisms and plasma lipid levels. Our results are consistent with several previous studies that provided evidence of the association between these gene variants and serum lipid levels.

Abnormal blood lipid profiles are influenced by multiple genes and environmental factors, each of which can contribute a minor marginal effect. To further assess their combined effects, gene-gene and gene-environment interactions were explored using GMDR, which is based on the score of a generalized linear model and is a special case of the original MDR method [32]. MDR has proven to be a useful statistical tool for detecting gene-gene interactions while avoiding ‘‘the dimension curse” [36]. However, existing MDR approaches do not allow for covariates. The GMDR, which is based on the MDR, allows the adjustment of discrete and quantitative covariates and is applicable to both dichotomous and continuous data [46]. Given that the GMDR method is immune to bias and invalidity in the presence of population heterogeneity [47], it provides greater flexibility for a population-based study design [32] and is therefore highly useful for revealing genetic architecture in terms of gene-gene and gene-environment interactions that are responsible for dyslipidemia.

In the present study, results of the GMDR analysis for the gene-gene interactions showed that the combination APOA1rs670-ABCA1rs1800976-ABCA1rs4149313-LCATrs1109166 was the best model irrespective of whether a dichotomous (sex, smoking, and drinking habits) or continuous (age and blood pressure) variable was measured after adjusting for covariates; this suggests that these four variants together are associated with the etiology of dyslipidemia. For the gene-environment analysis adjusted for covariates, including age, sex, and blood pressure, the five-factor interaction model, ABCA1rs1800976-ABCA1rs4149313-LCATrs1109166-obesity-smoking, was determined to be the best model for dyslipidemia. Results indicated an interaction comprising ABCA1rs1800976-ABCA1rs4149313-LCATrs1109166-obesity-smoking, which could have influenced dyslipidemia outcomes. Thus, our findings agree with previous reports showing that dyslipidemia is a complex trait caused by multiple environmental and genetic factors and their interactions [48–50]. Conflicting results were obtained from studies that focused on the association of single gene polymorphisms in genes involved in RCT and other risk factors with lipid levels [13–15, 20, 21, 23–26]. Thus, the contributions of gene-gene and gene-environment interactions to dyslipidemia can provide a more mechanistic explanation for this condition.

Results of haplotypes analyses using SHEsis software regarding the association of APOA1, ABCA1, and LCAT haplotypes and dyslipidemia showed that the A-C-A-C-A haplotype was significantly more frequent in the case group than in the control group, whereas the G-G-G-T-G haplotypes was less frequent in the case group than in the control group. The results indicated that the A-C-A-C-A haplotype can serve as a risk factor for dyslipidemia, whereas the G-G-G-T-G haplotype can serve as a protective factor against dyslipidemia.

Admittedly, the present study has certain limitations. First, serum lipid levels are regulated by multiple environmental and genetic factors and their interactions. However, we only investigated the interactions of five SNPs located in three genes and several environmental factors on dyslipidemia. Other environmental and genetic factors and their interactions were excluded; thus, “missing heritability” is still expected. Second, the current study was conducted in Xinjiang national region, which is located in the remote western region of China. No independent replication studies on the interactions between SNPs and environmental factors influencing dyslipidemia have been conducted for this population. Thus, the observed associations and interactions between these SNPs and dyslipidemia in the studied population may not represent the major characteristics of this conditions in other population groups. Elucidating epistasis in multidimensional space to infer gene function remains an interpretative challenge. It may be more practical to derive biological explanations for epistasis by investigating the physiological effects of the target polymorphisms [46, 51].

## Conclusions

Our findings showed that the APOA1rs670 and LCATrs2292318 polymorphisms are associated with dyslipidemia. The gene-gene interaction model comprising APOA1rs670-ABCA1rs1800976-ABCA1rs4149313-LCATrs1109166 and the gene-environment interaction model comprising ABCA1rs1800976-ABCA1rs4149313-LCATrs1109166-obesity-smoking were found to be the best predictive models for dyslipidemia. In addition, the A-C-A-C-A haplotype can act as a risk factor for dyslipidemia, whereas the G-G-G-T-G haplotype can serve as a protective factor against dyslipidemia.

## Supporting information

S1 File (XLSX)
